# Autophagy in Osteoarthritis: A Double-Edged Sword in Cartilage Aging and Mechanical Stress Response: A Systematic Review

**DOI:** 10.3390/jcm13103005

**Published:** 2024-05-20

**Authors:** Dong-Yeong Lee, Md Entaz Bahar, Chang-Won Kim, Min-Seok Seo, Myung-Geun Song, Sang-Youn Song, Soung-Yon Kim, Deok-Ryong Kim, Dong-Hee Kim

**Affiliations:** 1 Department of Orthopaedic Surgery, Barun Hospital, Jinju 52725, Republic of Korea; whatttary@hanmail.net; 2 Department of Biochemistry and Convergence Medical Sciences, Institute of Health Sciences, College of Medicine, Gyeongsang National University, Jinju 52727, Republic of Korea; entazbahar@gnu.ac.kr (M.E.B.); majestyno1@naver.com (M.-S.S.); 3 Department of Orthopaedic Surgery, Institute of Medical Science, Gyeongsang National University Hospital, Jinju 52727, Republic of Korea; kcw_blue@naver.com (C.-W.K.); songsangyoun@gmail.com (S.-Y.S.); soungyon.kim@gmail.com (S.-Y.K.); 4 Department of Orthopaedic Surgery, Inha University Hospital, Incheon 22212, Republic of Korea; piano10000@naver.com

**Keywords:** chondrocyte, cartilage, arthritis, osteoarthritis, autophagy

## Abstract

**Background**: Although osteoarthritis (OA) development is epidemiologically multifactorial, a primary underlying mechanism is still under debate. Understanding the pathophysiology of OA remains challenging. Recently, experts have focused on autophagy as a contributor to OA development. **Method**: To better understand the pathogenesis of OA, we survey the literature on the role of autophagy and the molecular mechanisms of OA development. To identify relevant studies, we used controlled vocabulary and free text keywords to search the MEDLINE, EMBASE, the Cochrane Central Register of Controlled Trials, Web of Science, and SCOPUS database. Thirty-one studies were included for data extraction and systematic review. Among these studies, twenty-five studies investigated the effects of autophagy in aging and OA chondrocytes, six studies examined the effects of autophagy in normal human chondrocytes, and only one study investigated the effects of mechanical stress-induced autophagy on the development of OA in normal chondrocytes. **Results**: The studies suggest that autophagy activation prevents OA by exerting cell-protective effects in normal human chondrocytes. However, in aging and osteoarthritis (OA) chondrocytes, the role of autophagy is intricate, as certain studies indicate that stimulating autophagy in these cells can have a cytotoxic effect, while others propose that it may have a protective (cytoprotective) effect against damage or degeneration. **Conclusions**: Mechanical stress-induced autophagy is also thought to be involved in the development of OA, but further research is required to identify the precise mechanism. Thus, autophagy contributions should be interpreted with caution in aging and the types of OA cartilage.

## 1. Introduction

Osteoarthritis (OA) is the most common form of arthritis and is an age-related degenerative disease that is becoming more prevalent due to human longevity [1]. Osteoarthritis (OA) is characterized by the gradual deterioration of articular cartilage, resulting in pain, stiffness, and diminished joint mobility [2]. Mechanical stress, such as aberrant joint loading or trauma, can activate degenerative processes, leading to degeneration [3]. Persistent inflammation in the joint worsens the deterioration of cartilage, with inflammatory cytokines playing a role in causing discomfort [4]. Metabolic variables such as obesity and dyslipidemia elevate the risk of osteoarthritis by stimulating joint inflammation and causing the breakdown of cartilage [5]. Genetic variations impact an individual’s vulnerability to osteoarthritis by altering the metabolism of cartilage and the occurrence of inflammation [6]. Chondrocyte dysfunction alters the balance of cartilage maintenance, resulting in higher breakdown of the cartilage matrix and reduced production [7]. Alterations in the synovium, such as inflammation, additionally leads to impaired joint function. Neural alterations, such as increased sensitivity of pain receptors, sustain discomfort associated with osteoarthritis [8]. In general, the development of osteoarthritis (OA) includes an intricate interaction between mechanical, inflammatory, metabolic, genetic, and neurogenic variables. Although the development of OA is epidemiologically multifactorial, age is one of the most significant factors. However, OA may develop in young people due to joint overuse, obesity, and post-traumatic injury. Conversely, older people harbor the same risk factors but do not develop the disease. Hence, a basic underlying mechanism is presumed but not yet proved.

The pathophysiology of OA is still challenging. Recently, many researchers have focused on autophagy as one of major contributors to cartilage injury and OA development [9,10]. Autophagy is a cellular homeostasis process that regulates energy metabolism as well as the removal of damaged and dysfunctional macromolecules and organelles [11]. Usually, autophagy plays a cytoprotective role. For instance, autophagy-related genes (ATG) knockdown accelerated rather than delayed cell death [12,13]. However, uncontrolled upregulation of autophagy leads to cell death. Under these conditions, autophagy-related apoptosis was activated, or cells were unable to survive the non-specific degradation of large amounts of cytoplasmic contents [14]. Many authors have reported that autophagy activity decreased with age [15,16]. Autophagy may exert cytoprotective effects in young cartilage, whereas upregulated autophagy can lead to autophagy-associated cell death in OA chondrocytes. These results suggest that autophagy plays both a cytoprotective and a death-promoting role in the pathogenesis of OA. Furthermore, the role of autophagy differs based on the stage and the layer of OA cartilage [15,17]. Almonte-Becerril et al. [17] demonstrated that, in the early stage, OA chondrocytes in the superficial zone showed increased autophagy activity to avoid cell death. In contrast, in the deep zone, the authors verified the absence of autophagy and promoted apoptosis. This may be associated with chondrocytes substitution and the abnormal calcification of the cartilage present in late-stage OA.

Autophagy functions as a quality control mechanism and is critical for cell response to stress [18]. However, autophagy acts as a double-edged sword (cytotoxic or cytoprotective) in different situations. These differences exist between animals and humans, even in the same type of chondrocytes. Moreover, autophagy can impact cells differently depending on the phase of the disease or the presence of mechanical stress [19]. Furthermore, when induced using the same agents, the outcome of autophagy varies by time and dose of induction [20]. Differences in cell type, the age of subjects, reagent concentrations, exposure time, genetics, and cell culture conditions may contribute to the differences in the autophagic response [15,17,20,21]. To address these issues, we conducted a comprehensive systematic review of studies on autophagy contributions in human chondrocyte biology as a new therapeutic target for cartilage injury and OA. We predominantly focused on verifying the regulatory mechanisms for controlling autophagy in human chondrocytes. The purpose of this review is to better understand the development and pathogenesis of OA.

## 2. Materials and Methods

### 2.1. Study Selection

Comprehensive databases were used to identify studies investigating the role of autophagy in human chondrocytes. The first search strategy was carried out on 23 February 2023, and it was revised on 22 November 2023. This study was based on the Cochrane Review methods. The reporting was in accordance with the preferred reporting items for systematic reviews and meta-analyses (PRISMA) statements (Tables S1 and S2). To identify relevant studies, the controlled vocabulary and free text words were used to search the MEDLINE, EMBASE, the Cochrane Central Register of Controlled Trials, Web of Science, and SCOPUS databases. The search string for our study included appropriate keywords about osteoarthritis, autophagy, cartilage aging, the mechanical stress response, etc. After obtaining our search strings, the subsequent step was to conduct searches using various combinations of these strings to pinpoint literature that precisely addressed parts of our research topic. This included specifying the keywords, Boolean operators, and any other search terms or filters utilized to identify relevant studies in electronic databases. Some examples included “autophagy” or “macroautophagy” or “chondrocyte autophagy” and “osteoarthritis” or “degenerative joint disease” and “cartilage aging” or “cartilage degeneration” and “mechanical stress” or “mechanotransduction” and “systematic review” or “review” or “meta-analysis”. We searched all relevant studies regardless of language, publication type (e.g., article, poster, conference article, and instructional course lecture), publication journal, and publication year. Reference lists of the identified studies were scrutinized to identify possible additional publications not found via electronic or manual searches.

### 2.2. Eligibility Criteria

Studies were included in this systematic review if they (1) investigated the effects of reagent-induced autophagy on normal, aging, or OA human chondrocytes; (2) investigated the effects of mechanical stress-induced autophagy on human chondrocytes; or (3) demonstrated a relationship between autophagy and the development of OA. However, studies were excluded if they (1) did not investigate OA but instead investigated rheumatoid arthritis or ankylosing spondylitis; (2) examined other cells, such as osteocytes, synovial fibroblasts, tenocytes, myocytes, nucleus pulposus, annulus fibrosus, spinal cord, and menisci; (3) used animal subjects, such as rats, mice, goats, and porcine; (4) investigated mechanisms not related to the development of OA; or (5) lacked an explanation of the relationship between autophagy and the development of OA. Moreover, in order to perform a thorough literature review that included a variety of relevant research regardless of their age, we decided not to limit inclusion based only on the publication date. The authors intend to comprehensively synthesize current information and evidence on the topic by expanding their research scope to include a wider range of literature, both older and more recent. In addition, the inclusion of older studies enabled the integration of a historical viewpoint, showcasing the progression of knowledge and comprehension of the subject matter throughout the years. This methodology allowed readers to track the evolution of ideas, approaches, and paradigms in the area, leading to a more thorough comprehension of the research landscape.

### 2.3. Extraction of Data

Two authors independently assessed the titles or abstracts of the studies identified via the query terms and then appraised the full papers. Final inclusions were determined through discussion and consensus. The eligible data were independently abstracted into predefined formats and checked for accuracy by the investigators. These included defining precise inclusion and exclusion criteria, utilizing thorough research techniques, keeping an eye out for duplicates, utilizing reference management software, assessing conference proceedings independently, cross-referencing citations, keeping open records of the search process, and guaranteeing unique outcomes by scrutinizing methodology and research designs. We chose to utilize versatile software applications such as Microsoft 365 (Microsoft Corporation, services using gnu.ac.kr email addresses) or Google Sheets (Google LLC, Mountain View, CA, USA) for the purpose of extracting data, taking into consideration the intricacy of the evaluation and the resources at hand. We employed EndNote (Endnote ^TM^ 21, Philadelphia, PA, USA), a reference management software that facilitates the organization and annotation of research records during the data extraction process. In addition, we devised personalized variables and tags to extract pertinent data from each specific study using EndNote.

### 2.4. Characteristics of the Included Studies

**Figure 1:**
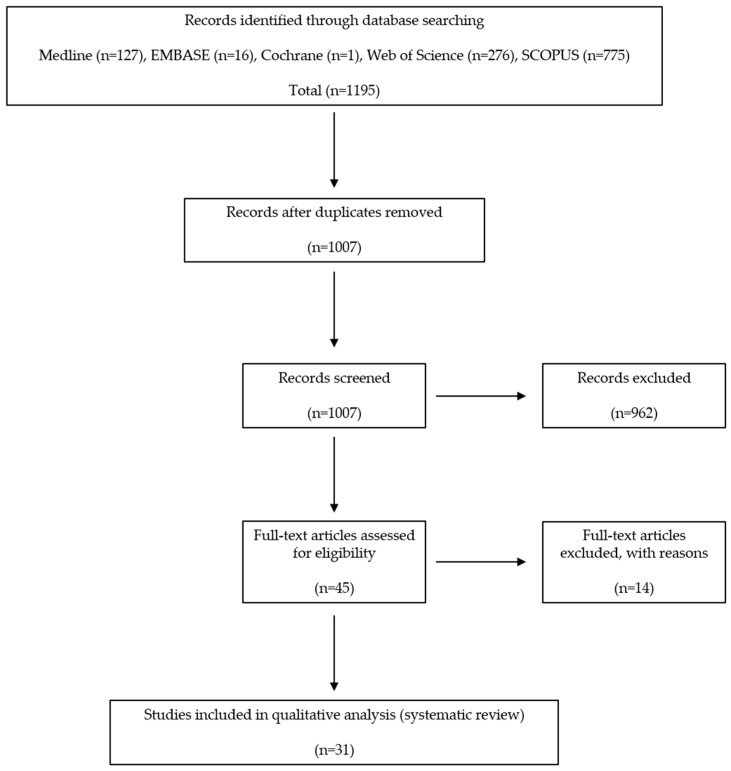
Preferred reporting items of systematic reviews and meta-analysis (PRISMA) flow diagram.

We identified a total of 1195 relevant articles. Of these, 188 were duplicates. We screened the remaining 1007 using titles and abstracts. All but 45 were excluded, because they were irrelevant to the purpose of the present study. A full-text review was performed for these 45 articles, and 14 were excluded because they met the exclusion criteria. The exclusion criteria applied during the study selection process included factors such as study design, population characteristics, intervention types, outcome measures, and any other relevant factors that led to the exclusion of certain. Finally, 31 studies were included for data extraction and systematic review, all of which were written in English (Figure 1) [10,20,22,23,24,25,26,27,28,29,30,31,32,33,34,35,36,37,38,39,40,41,42,43,44,45,46,47,48,49,50].

## 3. Results

### 3.1. Study Selection

Information about the study and experimental characteristics (the authors, reagents, materials, and genes), the condition of the subject’s cartilage (normal or OA primary human chondrocytes cell line), the effect of the reagents in autophagy (activation or inhibition), the role of the reagent in osteoarthritis (induction or prevention), and related mechanisms were obtained (Table 1). The reagents may have a dual effect on OA pathogenesis. On the one hand, a condition where cells become less responsive to reagents is associated with metabolic dysregulation and contributes to the induction or exacerbation of OA. On the other hand, the reagents possess anti-inflammatory and anabolic effects on cartilage, which could potentially prevent or attenuate OA progression by promoting cartilage repair and reducing inflammation. This review covered thirty-one in vitro studies that were written in the English language (Figure 1). Among them, twenty-four studies were on OA chondrocytes (77.4%), five studies were on normal chondrocytes (16.1%), and one study was on both normal and OA chondrocytes (3.1%) and examined the role of autophagy in human chondrocytes. Furthermore, only one study investigated the effects of mechanical stress-induced autophagy in normal human chondrocytes (3.1%).

This analysis provides a thorough examination of the complex connection between the regulation of autophagy and the pathophysiology of osteoarthritis (OA), providing valuable insights for future research and the development of therapeutic interventions.

### 3.2. The Role of Autophagy in Aging and OA Chondrocytes

Twenty-five studies investigated the role of autophagy in aging and OA chondrocytes or the C28/I2 cell line in which OA was induced by IL-1β [22,23,24,25,26,27,28,29,30,32,33,34,35,36,37,39,40,41,43,44,45,46,47,48,49]. The included studies described inconsistent effects of autophagy on aging or OA chondrocytes. Twelve studies [28,30,32,33,34,35,36,37,40,41,45,47] reported that the activation of autophagy protected chondrocytes from OA. Consistent with those findings, six studies [22,25,26,46,48,49] reported that the inhibition of autophagy was cytotoxic to OA chondrocytes. Therefore, these results emphasized the role of autophagy in preventing the development of OA. Conversely, the remaining seven studies [23,24,27,29,39,43,44] showed that the activation or inhibition of autophagy was associated with the development or prevention of OA, respectively, suggesting that autophagy activation could also lead to autophagy-dependent cell death in OA chondrocytes.

The findings emphasize the intricate nature of autophagy’s involvement in the development of osteoarthritis and emphasize the necessity for additional research to clarify its exact processes and potential therapeutic applications.

### 3.3. The Role of Autophagy in Normal Human Chondrocytes

Six studies [10,20,31,36,38,42] examined autophagy in normal primary human chondrocytes and cell lines (C28/I2 or T/C 28a2). Five studies [10,31,36,38,42] reported that the activating autophagy prevented the development of OA. Another study, [17], revealed a biphasic effect of advanced glycation end-products (AGEs) on autophagy in a dose- and time-dependent manner. Low doses of AGEs over a short period of time stimulated chondrocyte proliferation and autophagy, whereas high doses and long exposure to AGEs inhibited cell viability and autophagy by regulating the Akt/mTOR signaling pathway. These observations also indicate that autophagy activation is cytoprotective to normal human chondrocytes. The general conclusion of the above six studies demonstrated that autophagy protects chondrocytes. Thus, the activation of autophagy could, once again, prevent the development of OA in normal human chondrocytes. Furthermore, one study, [36], reached similar conclusions in cell lines (C28/I2) and primary OA human chondrocytes.

The results indicate that stimulating autophagy is beneficial for the health of normal human chondrocytes and has the potential to hinder the progression of OA, especially in both C28/I2 cell lines and primary human chondrocytes from individuals with OA.

### 3.4. The Role of Mechanical Stress-Induced Autophagy in Normal Human Chondrocytes

One study, [50], investigated the effects of mechanical stress-induced autophagy on OA development in normal chondrocytes. Mechanical stress-induced autophagy was found to be related to the expression of microRNA-590-5p and transforming growth factor-β1 (TGFβ1). Mechanical pressure injury significantly increased the expression of microRNA-590-5p and decreased the expression of TGFβ1. Both changes led to the activation of autophagy and more apoptosis. The authors concluded that mechanical stress-induced autophagy was associated with the development of OA. Based on our findings, it has been concluded that autophagy induced by mechanical stress was associated with the progression of OA.

## 4. Discussion

The role of autophagy in OA-associated cartilage deterioration has been discussed in prior review articles [51,52,53]; however, the purpose of this study was to comprehensively consolidate recent advancements in this field, while also offering distinct perspectives and valuable insights. In the present systematic review, we assessed the evidence from in vitro studies investigating the effects of autophagy on human chondrocytes. This aspect of our analysis offers significant perspectives on new therapeutic approaches and emphasizes areas for future investigation and clinical intervention. Most of the included studies showed that the activation of autophagy was cytoprotective and prevented the development of OA in the chondrocyte cells [28,30,32,33,34,35,36,37,40,41,45,47]. However, some studies reported an opposing cytotoxic role of autophagy [22,25,26,46,48,49]. These studies varied in their approaches to modulating autophagy, leading to differing effects on OA progression depending on the specific conditions.

We deliberately chose to concentrate exclusively on chondrocytes in our research because of their pivotal function in preserving the structural integrity of articular cartilage, which is a crucial element in the progression of OA [54]. Gaining a comprehensive understanding of how autophagy is regulated in chondrocytes is crucial for uncovering the mechanisms behind OA [55]. While it is possible that autophagy modification in other cell types is relevant and significant to osteoarthritis, such as synoviocytes or osteoblasts, we did not study them due to little research and the extensive body of literature on chondrocytes [52]. Furthermore, the malfunction of chondrocytes plays a direct role in the deterioration of cartilage, highlighting their importance in the development of osteoarthritis [56]. Directing therapeutic efforts towards autophagy in chondrocytes shows potential for treating osteoarthritis. However, additional research is required to investigate the control of autophagy in other cell types that are implicated in OA, in order to achieve a thorough understanding of the genesis of OA. Recently, several review studies have been published that specifically explore the involvement of autophagy in the deterioration of cartilage associated with OA.

Generally, autophagy is a major physiological mechanism that targets altered and dysfunctional macromolecules, membranes, and organelles for delivery to lysosomes for degradation and recycling [57,58,59,60]. *Atg1* (ULK1 (unc-51 like autophagy activating kinase 1)), *Atg6* (Beclin 1), and *Atg8* (LC3) are three major regulators of autophagy. Age-related and mechanically induced OA in humans is associated with a reduction and loss of ULK1, Beclin1, and LC3 expression in articular cartilage, meaning decreased basal autophagy activity [16]. In certain conditions, this leads to a form of cell death that is characterized by cytoplasmic vacuolation, termed type II programmed cell death or cell death by autophagy [61,62,63]. These conditions include the age of the subjects, the stage of OA, reagent concentrations, exposure time to the stimuli, and the chondrocyte layer [15,17,20,21]. The emerging evidence from this systematic review of the literature points to a divergent role (cytoprotective or cytotoxic) of autophagy in chondrocytes and OA that depends on the experimental settings.

Autophagy affects chondrocyte viability and modulates OA-related gene expression in the extracellular matrix (ECM). Sasaki et al. [21] reported that reactive oxygen species (ROS) were generated from damaged mitochondria in OA cartilage; autophagy removed the damaged mitochondria, decreasing the ROS level to protect the chondrocytes. In addition, activated autophagy was observed in the superficial zone of the articular cartilage, and this response was regarded as an adaptive response of the cartilage to wear or mechanical stress. When non-traumatic cartilage injury occurs, the damage is observed first in the superficial zone in magnetic resonance imaging (MRI) or arthroscopy according to the Outerbridge classification system or International Cartilage Repair Society (ICRS) grading system [64,65]. Deceased basal autophagic activity in superficial cartilage renders it susceptible to age-related changes [16,21,29]. Reduced autophagy caused a reduction in *COL2A1* and *aggrecans*, which are genes expressing collagen and aggrecan, and an increase in cartilage-degrading enzymes MMP-13 and ADAMTS5, leading to the development of OA [21]. Goutas et al. [66] reported that the development of OA was associated with reduced autophagy and mitochondrial dysfunction. However, the upregulation of autophagy could lead to autophagic cell death, increasing the risk factor of OA. Thus, autophagy induction as a method to alleviate OA needs to be considered with caution. Therefore, whether autophagy is cytoprotective or cytotoxic to chondrocytes may depend on the stage of OA, how the autophagy is induced, and whether apoptosis is involved. Further research is required to answer these questions.

Mechanical stress induces autophagy. The ability to respond and adapt to mechanical challenges is crucial for cell survival. Excessive mechanical loading to articular cartilage damages the cells and ECM, but little is known about the signaling pathways involved or the physiological relevance of these pathways. Mechanical stress likely activates autophagy independently of the classical mTOR/Akt or AMPK signaling [67]. Wang et al. [50] reported that mechanical stress could cause the development of OA via autophagy activation. Furthermore, although the effect of mechanical stress was demonstrated using bovine cartilage, Carames et al. [68] reported on the mechanical injury-induced cell death and the loss of sulfated glycosaminoglycan (sGAG). The study demonstrated that mechanical stress to articular cartilage activated the early steps of autophagy, but this defense mechanism was insufficient to protect articular cartilage against damage. In addition, mechanical injury suppressed autophagy later, predominantly in the superficial zone, where most cell death occurs [68]. Consistent with the study by King et al. [67], autophagy may protect against cartilage injury through the rapamycin/mTOR pathway. Therefore, in order to prevent post-traumatic cartilage damage and attenuate the subsequent development of OA, precise mechanisms related to mechanical stress and autophagy need to be further studied.

Recent reports have verified the association of OA with metabolic syndrome [69,70,71,72,73]. Diabetes mellitus (DM, hyperglycemia) and OA interact at local and systemic levels. Local effects of ROS and AGEs were implicated in cartilage damage [74]. Studies included in the present review revealed that delphinidin, a potent antioxidant, and AGEs affect the viability of human chondrocyte cells via autophagy [10,20]. A literature review revealed that the use of insulin was also associated with the development of OA via autophagy and inflammatory reactions [22]. Thus, DM is a risk factor for OA. Moreover, ectopic lipid deposition in chondrocytes induced by dyslipidemia might also initiate OA development, exacerbated by deregulated cellular lipid metabolism in joint tissue. Saturated fat intake increases low-density lipoprotein (LDL) cholesterol and total cholesterol, resulting in dyslipidemia [75]. Sekar et al. [27] reported that some kinds of saturated fatty acids were associated with the induction of autophagy via the NF-κB signaling pathway; decreased early chondrogenic markers, such as ACAN, COL2, and SOX; significantly increased degenerative markers, such as MMP13, ADAMTS4, and ADAMTS5; and finally, the development of OA. Furthermore, fenofibrate, a drug used to treat dyslipidemia, had protective effects on OA by reducing senescence and inflammation via autophagy activation in both aging and OA chondrocytes [35]. Hypertension is also associated with OA through subchondral ischemia, which can compromise nutrient exchange into the articular cartilage and trigger bone remodeling [69,76,77,78]. Cartilage and subchondral bone calcification contribute to the development and progression of OA. Our review revealed that adiponectin regulated vascular calcification, and AdipoRon, an adiponectin receptor agonist, significantly alleviated the calcification of OA chondrocytes via activating autophagy through the AMPK/mTOR signaling pathway [28]. Furthermore, obesity-related factors, particularly mechanical stress, contribute to OA development by regulating autophagy and other pathways, such as miR-590-5p or TGFβ1 [50]. As such, DM, dyslipidemia, hypertension, and obesity can all act as risk factors for OA. Therefore, to understand OA as a kind of metabolic syndrome, the fundamental mechanisms of autophagy induced by each factor and verifying the precise mechanism of autophagy in chondrocytes should be demonstrated through further studies.

The present review reveals several limitations in the studies of autophagy in human chondrocytes. The majority of studies investigating chondrocyte behavior have used samples of normal, aged, or osteoarthritic (OA) cartilage. However, some studies obtained normal cartilage for their research either from commercially available chondrocyte cell lines [10,36,38,42] or harvested from tissue specimens [31,42] that appeared normal upon visual inspection or radiological imaging during autopsy. These normal samples serve as a comparison to understand the differences in chondrocyte behavior between normal and OA-affected conditions. Indeed, the impact of aging cannot be completely ruled out, even when considering these parallels. Many OA cartilage samples were obtained from OA knees during total knee arthroplasties, so the stage of disease progression that could affect autophagy activity was not considered. In other words, the quality of the human chondrocytes used in the study was not thoroughly considered. These limitations are important in interpreting the results of the studies, because autophagy can present differently in different conditions. Thus, further studies are expected to clarify these points. It is also known that mitochondria are closely associated with the autophagy–inflammation cell death axis in organismal aging [16,66,79,80,81]. Defective autophagy and mitochondrial dysfunction are features of aging-related cartilage degeneration. Genetic mutations or stress to cartilage can generate damaged mitochondria and ROS. ROS promotes cell death through the vicious cycles of mitochondrial collapse. In this setting, autophagy exerts cell-protective effects by removing damaged mitochondria, but this aspect was not considered in this study review. Further research should investigate the mechanism of the mitochondrial and autophagy–inflammation cell death axis.

As with other systematic reviews of in vitro research, this work has limitations. Specifically, it can be difficult to collect data from studies that frequently present their findings in different ways, especially when looking at designs that have a high or unclear risk of bias. Some data may be hard to collect, and not all publications may rely on the same information; therefore, the authors’ interpretations of the results of the research that were included in this analysis may not have been sufficient. Consequently, these extra limitations still exist in addition to the preclinical study limitations. Furthermore, critical appraisal plays a vital role in evaluating evidence, identifying biases, and assessing methodological quality in research. Regrettably, this review faced limitations in conducting a thorough critical assessment due to resource constraints. This lack of critical appraisal may introduce potential biases and methodological flaws in the included research, impacting the overall robustness of the synthesized evidence. To enhance the rigor of future reviews, it is recommended to incorporate comprehensive critical appraisal and systematic evaluation of the study design, methodological quality, and potential sources of bias for improved validity of the synthesized data.

Moreover, our studies primarily used human chondrocyte-derived cell lines, which are expected to fill in any gaps in the role autophagy plays in the development of OA in humans and animal models.

## 5. Future Perspectives

Autophagy is a possible target for therapeutic intervention in osteoarthritis. It provides opportunities to maintain the balance of cartilage, reduce inflammation, and maybe slow down the progression of the disease. By understanding the complex processes that cause autophagy to become disrupted in the development of osteoarthritis and utilizing the ability to control autophagy for therapeutic purposes, we may work towards developing more efficient treatments that enhance the well-being of persons affected by this common joint condition. However, in order to further our comprehension of the role of autophagy in OA and apply this knowledge to develop successful therapeutic options, it is necessary to explore many paths for additional inquiry. Future research should prioritize investigating the precise mechanisms that cause autophagy dysregulation at various stages of osteoarthritis (OA) progression. Additionally, efforts should be made to identify new targets for autophagy that have potential for therapeutic use and to explore new methods for controlling autophagy in a tissue-specific and time-dependent manner. Moreover, utilizing cutting-edge technologies like single cell sequencing and omics profiling shows potential for discovering novel understandings of the intricate relationship between autophagy, cellular metabolism, and the development of osteoarthritis. Eventually, multidisciplinary teams mixing basic science research, preclinical models, and clinical trials are needed to bring autophagy-targeted therapies from the lab to the bedside and meet the unmet medical needs of OA patients.

## 6. Conclusions

**Figure 2:**
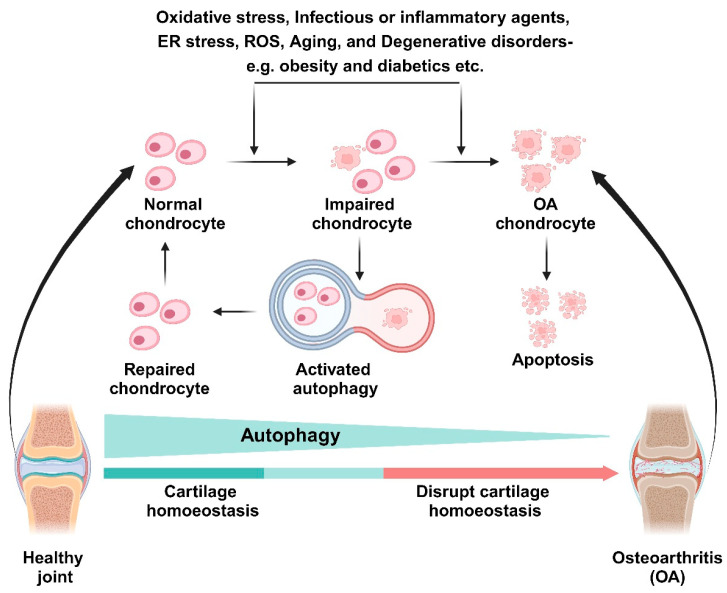
A schematic diagram of interconnecting autophagy and OA. Disruption of cartilage homeostasis by oxidative stress, infectious or inflammatory agents, ROS, aging, or degenerative diseases contribute to OA progression. Activated autophagy protects impaired chondrocytes and repairs them to healthy cells.

Autophagy activation is thought to prevent OA by exerting cell-protective effects in normal human chondrocytes (Figure 2). However, autophagy in aging and OA chondrocytes has various effects (cytotoxic or cytoprotective) in human chondrocyte cells. Thus, the effects of autophagy in aging and OA cartilage must be cautiously interpreted. Mechanical stress-induced autophagy is thought to be involved in the development of OA through autophagy or other mechanisms, necessitating further research. Therefore, to use autophagy as a new therapeutic target for OA, the exact mechanisms enabling autophagy to act in a cytoprotective role must be identified.

## Figures and Tables

**Table 1:** Effects of the pharmacological agents manipulating autophagy on human chondrocytes.

Study	Reagents	Condition of Subject’s Cartilage	Act of Reagents in Autophagy	Role of Reagents in Osteoarthritis	Related Mechanisms
Ribeiro et al. [22]	Insulin	OA human chondrocyte	Inhibition	Induction	Autophagy via Akt/mTOR signaling pathway. Activation of inflammation (loss of proteoglycans, increased MMP-13 and IL-1β).
Hwang et al. [23]	Monosodium urate (MSU)	OA human chondrocyte	Activation	Induction	Autophagy via Akt/mTOR signaling pathway. Independent of other cell death mechanisms, including apoptosis, ER stress-induced death, necroptosis, and pyroptosis.
Shen et al. [24]	Oxidized low density lipoprotein (Ox-LDL)	OA human chondrocyte	Activation	Induction	Increased ox-LDL and lectin-like ox-LDL receptor-1 (LOX-1) expression level was found in OA patients. Tumor necrosis factor-α mediated chondrocyte death.
Li et al. [25]	Plant homeodomain finger protein 23 (PHF 23)	OA human chondrocyte	Inhibition	Induction	Expression of PHF 23 increased in OA and was induced by IL-1β through inflammatory stress. PHF 23 suppressed autophagy of chondrocytes and accelerated apoptosis.
Wangyang et al. [26]	P63	OA human chondrocyte	Inhibition	Induction	P63 was overexpressed in OA patients. P63 upregulation correlated with suppressed autophagy and cell viability.
Sekar et al. [27]	Saturated fatty acid (palmitic acid (PA), stearic acid (SA), lauric acid (LA), myristic acid (MA))	OA human chondrocyte	PA, SA, MA: Autophagy activation LA: Similar to control	PA, SA, MA: OA inductionLA: Similar to control	Autophagy via NF-κB signaling pathway. PA, SA, MA treatment significantly decreased early chondrogenic markers such as ACAN, COL2, and SOX9 and significantly increased degenerative markers such as MMP13, ADAMTS4, and ADAMTS5. LA showed similar expressions in comparison with the controls.
Lee et al. [10]	Delphinidin	Normal human chondrocytes cell line (C28/I2),	Activation	Prevention	Delphinidin inhibited oxidative stress-induced apoptosis while it activated autophagy via NF-κB and Nrf2 pathways.
Duan et al. [28]	AdipoRon	OA human chondrocyte	Activation	Prevention	AdipoRon significantly alleviates the calcification of OA chondrocytes via activating AMPK/mTOR signaling to promote autophagy.
Xiao et al. [29]	Morroniside	OA human chondrocyte	Inhibition	Prevention	Morroniside inhibited chondrocyte autophagy through PI3K/AKT/mTOR signaling, thus it prevented cell death. Overexpression of autophagy enhanced the protection of Morroniside on chondrocytes.
Khan et al. [30]	Sucrose	OA human chondrocyte	Activation	Prevention	Sucrose induced autophagy in vitro dependent on the activation of AKT/mTOR/P70S6K signaling pathway and independent of ROS. Sucrose activated autophagy blocked IL-1β induced apoptosis and mRNA expression of MMP-13, COX-2, PGE-2, and IL-6.
Wang et al. [20]	Advanced glycation end products (AGEs)	Normal human chondrocyte	Biphasic effects	Biphasic effects	Low doses of AGEs over a short amount of time stimulated chondrocyte proliferation and autophagy by limiting phosphorylation of Akt/mTOR signaling. High dose and long exposure to AGEs inhibited cell viability and autophagy by increasing phosphorylation of Akt/mTOR signaling. AGEs can downregulate PPARG and that PPARG maintains cell viability by activating the Akt/mTOR signaling pathway as well as inducing autophagy.
Carames et al. [31]	Glucosamine	Normal human chondrocyte	Activation	Prevention	Glucosamine is an effective autophagy activator, and the enhancement of autophagy was mainly dependent on the Akt/FOXO and mTOR pathway.
Ansari et al. [32]	Butein	OA human chondrocyte	Activation	Prevention	Butein increased the phosphorylation of AMPKα^Thr-172^, TSC^Ser-1387^, and ULK1^Ser-317^ and inhibited the phosphorylation of mTOR^Ser-2448.^ Increased autophagy flux that correlated with the suppression of the IL-1β mediated expression of IL-6.
Liu et al. [33]	Astragaloside Ⅳ (AST)	OA human chondrocyte	Activation	Prevention	AST-mediated autophagy protected against chondrocyte apoptosis induced by IL-1β.
Moussa et al. [34]	Platelet rich plasma (PRP)	OA human chondrocyte	Activation	Prevention	PRP increased significantly the proliferation of chondrocytes, decreased apoptosis and increased autophagy via FOXO1, FOXO3, HIF-1. PRP caused a significant decrease in MMP3, MMP13, ADAMTS-5, IL-6, and COX-2 while increasing TGF-β, aggrecan, collagen type 2, TIMPs and intracellular IL-4, IL-10, IL-13.
Nogueira-Recalde et al. [35]	Fenofibrate (FN)	OA and ageing human chondrocyte	Activation	Prevention	FN (PPARα agonist) reduced proteoglycan loss and protected against cartilage degradation. PPARα was mainly expressed in the superficial zone in non-OA cartilage with decreased expression in OA patients. FN reduced both senescence and inflammation and increased autophagy in both aging human and OA chondrocytes.
Cetrullo et al. [36]	Hydroxytyrosol (HT)	Normal human chondrocytes cell line (C28/I2), OA human chondrocyte	Activation	Prevention	The protective effect requires the deacetylase sirtuin 1 (SIRT-1) and silencing of this enzyme prevented HT from promoting the autophagic process and cell survival. HT supports autophagy even in a SIRT-1-independent manner, by increasing p62 transcription, required for autophagic degradation of polyubiquitin-containing bodies. HT exerts its cell protective action in C28/I2 line and human OA chondrocytes by the same modalities.
Ansari et al. [37]	Butea monosperma (Butein, BME)	OA human chondrocyte	Activation	Prevention	BME activated autophagy via inhibition of mTOR pathway. BME suppressed the IL-1β induced expression of IL-6, MMP-3, MMP-9, and MMP-13.
Yu et al. [38]	T-2, HT-2 toxin	Normal human chondrocytes cell line (C28/I2)	Activation	Prevention	T-2 and HT-2 toxins induce apoptosis and autophagy, and the level of oxidative stress plays an important role in autophagy activation. The expression levels of apoptosis and autophagy induced by T-2 toxin were significantly higher when compared with those levels induced by the HT-2 toxin. The activation of autophagy can reduce oxidative damage and apoptosis.
Liao et al. [39]	Resveratrol (Sirt I inducer)	OA human chondrocyte	High levels of Sirt I inhibited autophagy in OA	Prevention	Sirt I regulates autophagy by interacting with Atg7. The expression of Sirt I might be age-related: it is high in young people and decreased in elderly and OA patients. Of note, the high levels of Sirt I reduced autophagy in OA. Through increasing the activity of Sirt I the autophagic cell death of OA chondrocyte could be inhibited.
Zhong et al. [40]	miRNA-335-5p	OA human chondrocyte	Activation	Prevention	The expression of miRNA-355-5p was significantly lower in OA chondrocytes. miRNA-355-5p can significantly alleviate inflammation in human OA chondrocytes by activating autophagy.
Wang et al. [41]	miR-140-5p/miR-149	OA human chondrocyte	Activation	Prevention	The overexpression of miR-140-5p/miR-149 inhibited apoptosis and promoted proliferation and autophagy of primary human chondrocytes via downregulating FUT1.
D’Adamo et al. [42]	miRNA-155	Normal human chondrocyte and cell line (T/C28a2)	Inhibition	Induction	miRNA-155 regulates autophagy by suppressing MAP1LC3, GABARAPL1, Atg3, Atg5, Atg, 14, ULK1, and FOXO3.miRNA-155 inhibited autophagy by activating mTOR pathway.
Yang et al. [43]	miRNA-411	OA induced C28/I2 chondrocyte cell line by IL-1β	Mimic: inhibition, Inhibitor: activation	Mimic: prevention, Inhibitor: induction	miRNA-411 regulates autophagy by targeting HIF-1α. miRNA-411 mimic inhibited autophagy by reducing HIF-1α while miRNA-411 inhibitor activated autophagy by increasing HIF-1α in chondrocytes. miRNA-411 was downregulated in OA chondrocyte, so this causes activation of autophagy by increasing HIF-1α. Thus, downregulated miRNA-411 is closely associated with the development of OA.
Tian et al. [44]	upregulated small nuclear RNA host gene 7 (SNHG7), downregulated miR-34a-5p	OA human chondrocyte	Inhibition	Prevention	SNHG7 and SYVN1 were downregulated, but miR-34a-5p was upregulated in OA. Upregulated SNHG7 promoted cell proliferation as well as inhibited cell apoptosis and autophagy by sponging miR-34a-5p through regulating SYNV1 in OA cells.
Yang et al. [45]	Long non-coding RNA reprogramming (lncRNA-ROR)	OA human chondrocyte	Activation	Prevention	Level of lncRNA-ROR was decreased in OA. Overexpression of lncRNA-ROR dramatically promoted cell viability of OA chondrocytes. Knockdown lncRNA-ROR inhibited apoptosis and promoted autophagy by regulating HIF-1α and p53.
Akasaki et al. [46]	Downregulated FOXO transcription factors	OA human chondrocyte	Inhibition	Induction	Reduced expression of FOXO transcription factors in chondrocytes increased susceptibility to cell death induced by oxidative stress. This was associated with reduced antioxidant proteins and autophagy-related proteins. FOXO downregulation: ADAMTS-4↑ and Chemerin↑ in OA chondrocytes.
Huang et al. [47]	Knockdown of serum- and glucocorticoid-regulated kinase 1 (SGK1)	OA human chondrocyte	Activation	Prevention	SGK1 was upregulated in OA cartilage. SGK1 knockdown leads to increased autophagy, subsequently inhibiting OA by regulating FOXO1. SGK1 knockdown: collagen II↑, aggrecan↑, ADAMTS-5↓, MMP-13↓, disintegrin↓
Hwang et al. [48]	29 kDa fibronectin fragment (29 kDa FN-f)	OA human chondrocyte	Inhibition	Induction	HMGB1 level was significantly lower in human OA cartilage. 29-kDa FN-f inhibits chondrocyte autophagy by modulating the mTOR pathway and HMGB1 signaling pathway.
He et al. [49]	Hox transcript antisense intergenic RNA (HOTAIR)	OA human chondrocyte	Inhibition	Induction	Upregulation of HOTAIR and downregulation of miR130a-3p were found in OA. Expression of HOTAIR resulted in apoptosis events caused by the sponging of miR-130a-3p to suppress autophagy, subsequently induced OA.
Wang et al. [50]	Upregulated microRNA-590-5p (miR-590-5p)	Normal human chondrocyte	Activation	Induction	Mechanical pressure injury resulted in a significantly increased expression of miR-590-5p and decreased expression of TGFβ1. The miR-590-5p targets TGFβ1 to regulate chondrocyte apoptosis and autophagy in response to mechanical pressure injury. Decreased miR-590-5p leads to increased cell viability and decreased autophagy. And, decreased TGFβ1 leads to increased autophagy and apoptosis.

OA, osteoarthritis; mTOR, mammalian target of rapamycin; MMP, matrix metalloproteinase; IL, interleukin; ER, endoplasmic reticulum; NF-κB, nuclear factor-κB; ACAN, aggrecan; COL2, type II collagen; SOX9, SRY (sex determining region Y) box 9; ADAMTS, a disintegrin and metalloproteinase with thrombospondin motifs; Nrf2, nuclear factor (erythroid-derived 2)-like 2; AMPK, 5′-adenosine monophosphate-activated protein kinase; PI3K, phosphoinositide 3-kinase; ROS, reactive oxygen species; COX, cyclooxynenase; PGE, prostaglandin E; PPARG, peroxisome proliferator-activated receptor-γ; FOXO, forkhead-box class O; TSC, tuberous sclerosis complex; ULK1, unc-51 like autophagy activating kinase 1; HIF-1, hypoxia-inducible factor-1; TGF-β, transforming growth factor-β; TIMPs, tissue inhibitors of metalloproteinases; Atg, autophagy-related gene miRNA, mi-croRNA; FUT1, fucosyltransferase 1; MAP1LC3, microtubule-associated protein 1 light chain 3; GABARAPL1, gamma-aminobutyric acid receptor-associated protein-like 1; SYNV1, synoviolin1; HMGB1, high-mobility group protein box 1.

## Data Availability

All relevant data are within the paper.

## Supplementary Materials

The following supporting information can be downloaded at: https://www.mdpi.com/article/10.3390/jcm13103005/s1, Table S1: PRISMA 2020 Checklist [82]; Table S2: PRISMA 2020 Checklist (Absctract) [82].

## Abbreviations

OA, osteoarthritis; mTOR, mammalian target of rapamycin; MMP, matrix metalloproteinase; IL, interleukin; ER, endoplasmic reticulum; NF-κB, nuclear factor-κB; ACAN, aggrecan; COL2, type II collagen; SOX9, SRY (sex determining region Y) box 9; ADAMTS, a disintegrin and metalloproteinase with thrombospomdin motifs; Nrf2, nuclear factor (erythroid-derived 2)-like 2; AMPK, 5′-adenosine monophosphate-activated protein kinase; PI3K, phosphoinositide 3-kinase; ROS, reactive oxygen species; COX, cyclooxygenase; PGE, prostaglandin E; PPARG, peroxisome proliferator-activated receptor-γ; FOXO, forkhead-box class O; TSC, tuberous sclerosis complex; ULK1, unc-51 like autophagy activating kinase 1; HIF-1, hypoxia-inducible factor-1; TGF-β, transforming growth factor-β; TIMPs, tissue inhibitors of metalloproteinases; Atg, autophagy related gene; miRNA, microRNA; FUT1, fucosyltransferase 1; MAP1LC3, microtubule-associated protein 1 light chain 3; GABARAPL1, gamma-aminobutyric acid receptor-associated protein-like 1; SYNV1, synoviolin 1; HMGB1, high-mobility group protein Box 1.
