# Supplementary material for: Autophagy in Osteoarthritis: A Double-Edged Sword in Cartilage Aging and Mechanical Stress Response: A Systematic Review
Source: J Clin Med. 2024 May 20;13(10):3005. doi: 10.3390/jcm13103005 (PMC11122125; doi:10.3390/jcm13103005)
Supplement: Supplementary file 1 [file jcm-13-03005-s001.zip › jcm-2979678-supplementary_2.pdf]

Journal of
Clinical Medicine

MDPI

Supplementary Materials

Section and Topic	Item #	Checklist item	Location where item is reported
**TITLE**			
Title	1	Identify the report as a systematic review.	Yes
**Background**			
Objectives	2	Provide an explicit statement of the objective(s) or question(s) the review addresses.	Yes
**METHODS**			
Eligibility criteria	3	Specify the inclusion and exclusion criteria for the review and how studies were grouped for the syntheses.	No
Information sources	4	Specify all databases, registers, websites, organisations, reference lists and other sources searched or consulted to identify studies. Specify the date when each source was last searched or consulted.	yes
Included studies	5	Give the total number of included studies and participants and summarise relevant characteristics of studies.	yes
Reporting bias assessment	6	Describe any methods used to assess risk of bias due to missing results in a synthesis (arising from reporting biases).	No
Certainty assessment	7	Describe any methods used to assess certainty (or confidence) in the body of evidence for an outcome.	No
**RESULTS**			
Synthesis of results	8	Present results for main outcomes, preferably indicating the number of included studies and participants for each. If meta-analysis was done, report the summary estimate and confidence/credible interval. If comparing groups, indicate the direction of the effect (i.e. which group is favoured).	Yes
**DISCUSSION**			
Discussion	9a	Provide a general interpretation of the results in the context of other evidence.	Yes
	9b	Discuss any limitations of the evidence included in the review.	Yes

Section and Topic	Item #	Checklist item	Location where item is reported
	9c	Discuss any limitations of the review processes used.	Yes
	9d	Discuss implications of the results for practice, policy, and future research.	Yes
**OTHER INFORMATION**			
Funding	10	Specify the primary source of funding for the review.	Yes
Competing interests	11	Declare any competing interests of review authors.	

From: Page MJ, McKenzie JE, Bossuyt PM, Boutron I, Hoffmann TC, Mulrow CD, et al. The PRISMA 2020 statement: an updated guideline for reporting systematic reviews. BMJ 2021;372:n71. doi: 10.1136/bmj.n71. For more information, visit: http://www.prisma-statement.org/.
